# Supplementary material for: The m6A methyltransferase METTL3 regulates muscle maintenance and growth in mice
Source: Nat Commun. 2022 Jan 10;13:168. doi: 10.1038/s41467-021-27848-7 (PMC8748755; doi:10.1038/s41467-021-27848-7)
Supplement: Supplementary file 1 — Supplementary Information [file 41467_2021_27848_MOESM1_ESM.pdf]

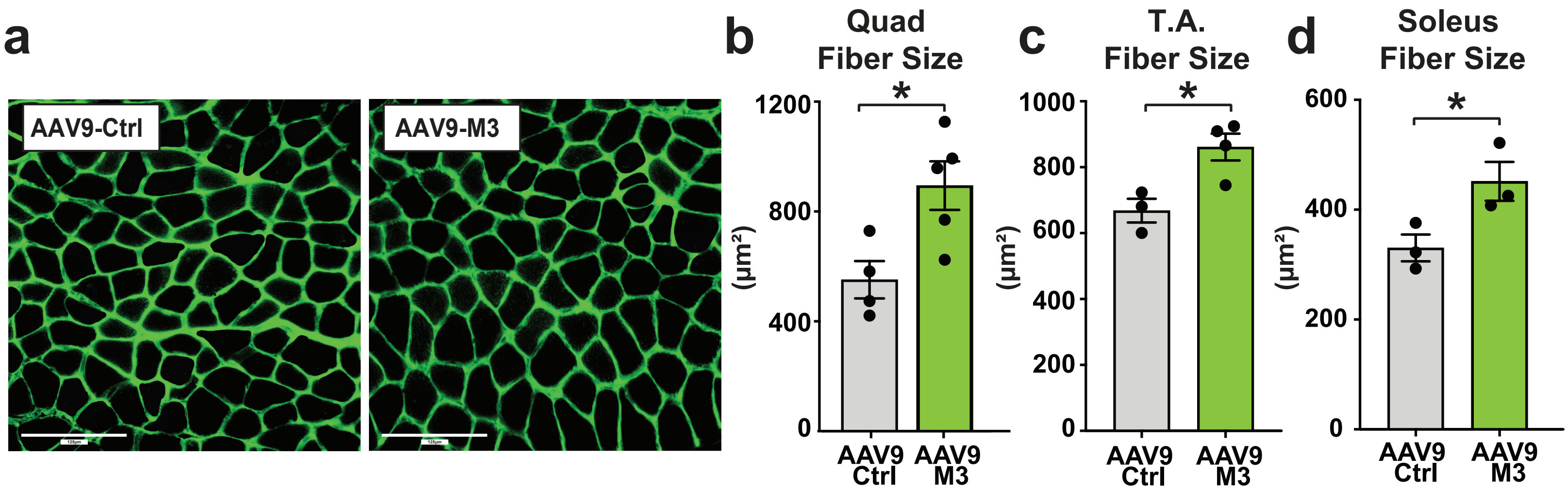

Supplementary Figure 1: Delivery of systemic AAV9-METTL3 to neonatal mice. (a) representative wheat germ agglutinin (WGA; green) stained plantaris images 8 weeks after AAV injection in AAV9-Ctrl or AAV9-M3 animals. (b-d) Myofiber size quantification from quadriceps (quad), tibialis anterior (T.A.), and soleus in animals treated with AAV9-Control or AAV9 overexpressing METTL3. Biological animal replicates: n= 4 (AAV9-Ctrl) and 5 (AAV9-M3) in panel b; n= 3 (AAV9-Ctrl) and 4 (AAV9-M3) in panel c; n= 3 per group in panel d. Data are represented as mean ± SEM. * p ≤ 0.05, by 2-sided Student’s t test. Scale bar = 125μm. Source data are provided as a Source Data file.

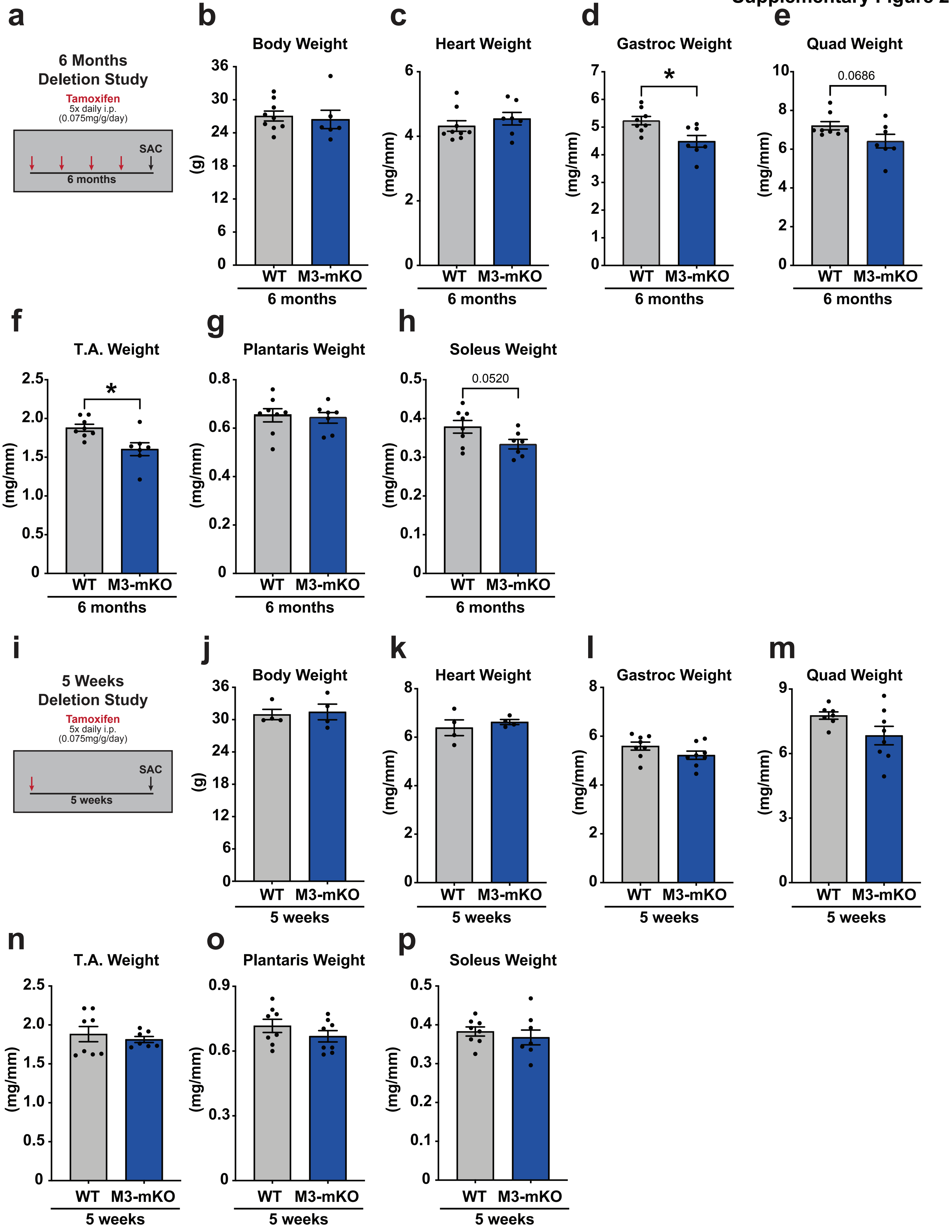
a
6 Months Deletion Study
Tamoxifen
5x daily i.p.
(0.075mg/g/day)
SAC
6 months
b
Body Weight
(g)
WT
M3-mKO
6 months
c
Heart Weight
(mg/mm)
d
Gastroc Weight
*
e
Quad Weight
0.0686
f
T.A. Weight
g
Plantaris Weight
h
Soleus Weight
0.0520
i
5 Weeks Deletion Study
5 weeks
j
k
l
m
n
o
p

Supplementary Figure 2: Deletion of Mettl3 in muscle for varying amounts of time. (a) Schematic of tamoxifen dosing and experimental plan for inducible Mettl3 deletion in muscle for 6 months. (b) Body, (c) heart, (d) gastrocnemius (gastroc), (e) quadriceps (quad), (f) tibialis anterior (T.A.), (g) plantaris, and (h) soleus muscle weight relative to tibia length in mice with conditional knockout of muscle Mettl3 or wild-type controls 6 months following initial tamoxifen dosing. (i) Schematic of tamoxifen dosing and experimental plan for inducible Mettl3 deletion in muscle for 5 weeks. (j) Body, (k) heart, (l) gastrocnemius, (m) quadriceps, (n) tibialis anterior, (o) plantaris, and (p) soleus muscle weight relative to tibia length in mice with conditional knockout of muscle Mettl3 or wild-type controls 5 weeks following initial tamoxifen dosing. Biological animal replicates: n= 9 (WT) and 6 (mKO) in panel b; n= 9 (WT) and 7 (mKO) in panel c; n= 8 (WT) and 7 (mKO) in panel d-h; n= 4 per group in panel j and k; n= 8 per group in panel l-p. Data are represented as mean ± SEM. * $p \leq 0.05$, by 2-sided Student's t test. Source data are provided as a Source Data file.

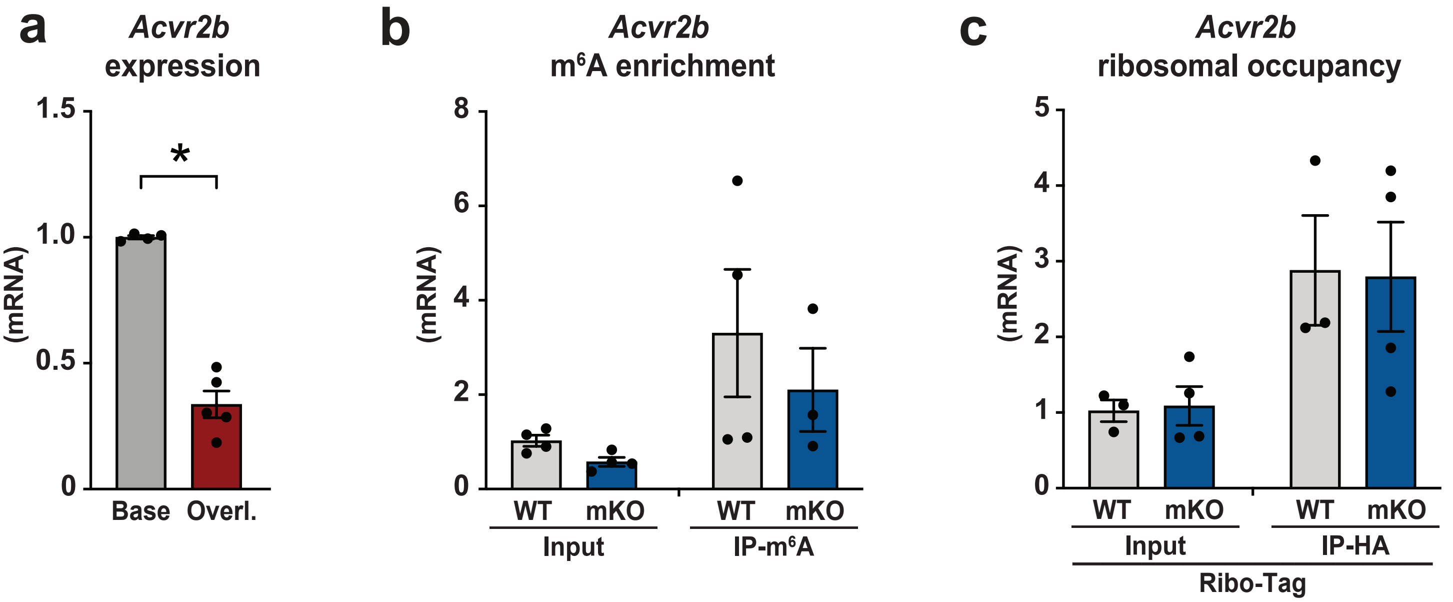

Supplementary Figure 3: Acvr2b is not regulated by METTL3-m6A Axis. (a) Relative expression of *Acvr2b* in sham or overloaded animals 7 days following surgery. (b) Relative m^6A enrichment on *Acvr2b*, determined by qPCR analysis, following m^6A immunoprecipitation in WT and M3-mKO plantaris muscles. (c) Relative ribosome occupancy enrichment, determined by qPCR analysis, of *Acvr2b* following Ribo-Tag immunoprecipitation in Ribo-Tag WT and M3-mKO plantaris muscles. Biological animal replicates: n= 4 (baseline) and 5 (overload) in panel a; n= 4 per group in panel b; n= 3 (WT) and 4 (mKO) in panel c. Data are represented as mean ± SEM. * $p \leq 0.05$, by 2-sided Student's t test. 2-way ANOVA with Tukey's HSD multiple-comparison test was used for comparison of the mean of WT and M3-mKO animal inputs and immunoprecipitations. Source data are provided as a Source Data file.

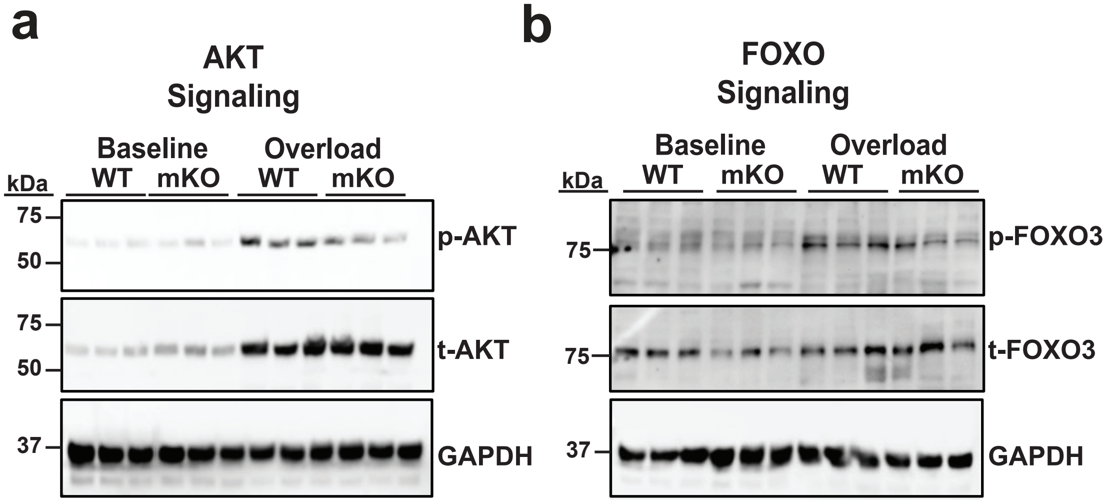

Supplementary Figure 4: Impact of the loss of METTL3 on AKT and FOXO signaling. (a) Western blot of phospho-AKT (p-AKT), total AKT (t-AKT), and GAPDH expression in muscles of WT and M3-mKO sham and overloaded mice 7 days following surgery. (b) Western blot of phospho-FOXO3 (p-FOXO3), total FOXO3 (t- total FOXO3), and GAPDH expression in muscles of WT and M3-mKO sham and overloaded mice 7 days following surgery. n = 3 per group for biological animal replicates. Source data are provided as a Source Data file.
