# Supplementary material for: The m6A methyltransferase METTL3 regulates muscle maintenance and growth in mice
Source: Nat Commun. 2022 Jan 10;13:168. doi: 10.1038/s41467-021-27848-7 (PMC8748755; doi:10.1038/s41467-021-27848-7)
Supplement: Supplementary file 3 — Description of Additional Supplementary Files [file 41467_2021_27848_MOESM3_ESM.docx]

Description of Additional Supplementary Files

**Title: Supplementary Data 1.**

Description: m^6^A modified transcripts found by meRIP-seq analysis on skeletal muscle.

**Title: Supplementary Data 2.**

Description: Ribo-seq and meRIP-seq cross analysis for ribo-enriched and m^6^A-modified transcript in WT and M3-mKO skeletal muscle.
