# Supplementary material for: The m6A methyltransferase METTL3 regulates muscle maintenance and growth in mice
Source: Nat Commun. 2022 Jan 10;13:168. doi: 10.1038/s41467-021-27848-7 (PMC8748755; doi:10.1038/s41467-021-27848-7)
Supplement: Supplementary file 6 — Reporting Summary [file 41467_2021_27848_MOESM6_ESM.pdf]

nature portfolio

Corresponding author(s): NCOMMS--21-19245A

Last updated by author(s): Nov 27, 2021

Reporting Summary

Nature Portfolio wishes to improve the reproducibility of the work that we publish. This form provides structure for consistency and transparency in reporting. For further information on Nature Portfolio policies, see our Editorial Policies and the Editorial Policy Checklist.

Please do not complete any field with "not applicable" or n/a. Refer to the help text for what text to use if an item is not relevant to your study.
For final submission: please carefully check your responses for accuracy; you will not be able to make changes later.

Statistics

For all statistical analyses, confirm that the following items are present in the figure legend, table legend, main text, or Methods section.

n/a	Confirmed	
☐	☒	The exact sample size (*n*) for each experimental group/condition, given as a discrete number and unit of measurement
☐	☒	A statement on whether measurements were taken from distinct samples or whether the same sample was measured repeatedly
☐	☒	The statistical test(s) used AND whether they are one- or two-sided<br>*Only common tests should be described solely by name; describe more complex techniques in the Methods section.*
☒	☐	A description of all covariates tested
☐	☒	A description of any assumptions or corrections, such as tests of normality and adjustment for multiple comparisons
☐	☒	A full description of the statistical parameters including central tendency (e.g. means) or other basic estimates (e.g. regression coefficient) AND variation (e.g. standard deviation) or associated estimates of uncertainty (e.g. confidence intervals)
☐	☒	For null hypothesis testing, the test statistic (e.g. *F*, *t*, *r*) with confidence intervals, effect sizes, degrees of freedom and *P* value noted<br>*Give P values as exact values whenever suitable.*
☒	☐	For Bayesian analysis, information on the choice of priors and Markov chain Monte Carlo settings
☒	☐	For hierarchical and complex designs, identification of the appropriate level for tests and full reporting of outcomes
☒	☐	Estimates of effect sizes (e.g. Cohen's *d*, Pearson's *r*), indicating how they were calculated

Our web collection on statistics for biologists contains articles on many of the points above.

Software and code

Policy information about availability of computer code

Data collection	Mice were single housed in open-circuit treadmills as part of the Comprehensive Lab Animal Monitoring System (CLAMS) and data was collected with OxyMax software 2.4.2 from Columbus Instruments. In vivo muscle force was collected using a muscle contractility apparatus, Model 1300A, from Aurora Scientific. Image collection was performed using an Invitrogen EVOS imaging system. Western blots were imaged using a BioRad ChemiDoc system. QPCR was performed using the BioRad CFX Connect. meRIP was sequenced using the Illumina HiSeq 2000 at the University of Chicago Genomics Facility. RiboSeq was sequenced with Complete Genomics-BGI Americas.
Data analysis	ImageJ was used for image quantification. ImageJ 1.49v and BioRad Image Lab 6.1 were used for western blot quantification. Microsoft excel version 16.16.24 and Biorad CFX Maestro were used for qPCR analysis. Bioinformatic analysis utilized using FASTQC (https://www.bio informatics.babraham.ac.uk/projects/fastqc/) and exomePeak2 analysis (https://github.com/ZW-xjtlu/exomePeak2), and executed using the Ohio State University Supercomputer, Webgestalt. GraphPad Prism 8 was used for graphing and statistical analysis.

For manuscripts utilizing custom algorithms or software that are central to the research but not yet described in published literature, software must be made available to editors and reviewers. We strongly encourage code deposition in a community repository (e.g. GitHub). See the Nature Portfolio guidelines for submitting code & software for further information.

Data

Policy information about availability of data

All manuscripts must include a data availability statement. This statement should provide the following information, where applicable:

- Accession codes, unique identifiers, or web links for publicly available datasets
- A description of any restrictions on data availability
- For clinical datasets or third party data, please ensure that the statement adheres to our policy

Data Availability: Data are provided through "Source Data" file and Gene Expression Omnibus submission GSE179368.

Field-specific reporting

Please select the one below that is the best fit for your research. If you are not sure, read the appropriate sections before making your selection.

☒ Life sciences ☐ Behavioural & social sciences ☐ Ecological, evolutionary & environmental sciences

Life sciences study design

All studies must disclose on these points even when the disclosure is negative.

Sample size	For image quantifications, sufficient cells were quantified per biological replicate to allow for a clear distribution. For animal experiments, sample sizes were selected based on the power calculations referenced within the IACUC animal protocol deemed to be appropriate and sufficient for realization of statistical significance in vivo experiments.
Data exclusions	No data were excluded from the analysis.
Replication	A minimum of 3 independent experiments were performed to reproduce each result.
Randomization	Randomization was performed using a random number generator. For animal experiments, M3-mKO mice were housed with their litter mate WT controls and cages/mice were randomly allocated to study groups based on order they were tagged for identification. Age-matched WT and mKO mice were randomly assigned to muscle overload, sham, vehicle, and/or inhibitor groups prior to any procedure.
Blinding	Generally, all experiments and measurements were performed in a blinded fashion. Investigators were blinded to mouse genotypes when performing ablation surgeries, and gravimetric data was collected with individuals weighing samples while blinded to genotypes. When performing muscle overload surgeries, the phenotypic difference between sham and ovreloaded animals was stark and obvious limiting the degree of blinding to this procedure, however the operator was blinded to the genotypes. Biochemical and bioinformatic approaches were performed in a blinded fashion.

Reporting for specific materials, systems and methods

We require information from authors about some types of materials, experimental systems and methods used in many studies. Here, indicate whether each material, system or method listed is relevant to your study. If you are not sure if a list item applies to your research, read the appropriate section before selecting a response.

Materials & experimental systems

n/a	Involved in the study	
☐	☒	Antibodies
☐	☒	Eukaryotic cell lines
☒	☐	Palaeontology and archaeology
☐	☒	Animals and other organisms
☒	☐	Human research participants
☒	☐	Clinical data
☒	☐	Dual use research of concern

Methods

n/a	Involved in the study	
☒	☐	ChIP-seq
☒	☐	Flow cytometry
☒	☐	MRI-based neuroimaging

Antibodies

Antibodies used	METTL3(1:2000, Abcam, #ab240595)<br>GAPDH (1:10,000, Fitzgerald Industries, #10R-G109a)<br>SMAD3 (#9523, Cell Signaling)<br>Phospho-SMAD3 (#9520, Cell Signaling)<br>Puromycin (#MABE343, EMD Millipore)

	Phospho-AKT (#4060, Cell Signaling)<br>AKT (#9272, Cell Signaling)<br>Phospho-FOXO3 (#9466, Cell Signaling)<br>FOXO3 (#2497, Cell Signaling)<br>YTHDF1 (#17479-AP, ProteinTech)<br>YTHDF2 (#ab220163, Abcam)<br>YTHDF3 (#sc-377119, Santa Cruz Biotechnology)
Validation	METTL3 validated in this publication through the use of M3-mKO<br>Puromycin validated in this publication using negative control-non puromycin injected<br>SMAD3 validation and publication references at Cell Signaling website<br>https://www.cellsignal.com/products/primary-antibodies/smad3-c67h9-rabbit-mab/9523<br>Phospho-SMAD3 validation and publication references at Cell Signaling website<br>https://www.cellsignal.com/products/primary-antibodies/phospho-smad3-ser423-425-c25a9-rabbit-mab/9520?site-search-type=Products&N=4294956287&Ntt=%239520&fromPage=plp&_requestid=3270955<br>Phospho-AKT validation and publication references at Cell Signaling website<br>https://www.cellsignal.com/products/primary-antibodies/phospho-akt-ser473-d9e-xp-rabbit-mab/4060?site-search-type=Products&N=4294956287&Ntt=%234060&fromPage=plp&_requestid=3271116<br>AKT validation and publication references at Cell Signaling website<br>https://www.cellsignal.com/products/primary-antibodies/akt-antibody/9272?site-search-type=Products&N=4294956287&Ntt=%239272&fromPage=plp&_requestid=3271270<br>Phospho-FOXO3 validation and publication references at Cell Signaling website<br>https://www.cellsignal.com/products/primary-antibodies/phospho-foxo3a-ser253-antibody/9466<br>FOXO3 validation and publication references at Cell Signaling website<br>https://www.cellsignal.com/products/primary-antibodies/foxo3a-75d8-rabbit-mab/2497?_=1635276090356&Ntt=2497&tahead=true<br><br>For antibodies against YTHDF1,YTHDF2, and YTHFD3 antibodies were used for mass spec, where the ability to IP the protein validated each antibody successfully.

Eukaryotic cell lines

Policy information about cell lines

Cell line source(s)	ATCC 3T3-L1 murine cell line
Authentication	Cells were purchased from ATCC and not further authenticated.
Mycoplasma contamination	Cells lines used were tested and confirmed negative for micoplasma contamination.
Commonly misidentified lines (See ICLAC register)	No commonly misidentified cell lines were used in the study.

Animals and other organisms

Policy information about studies involving animals; ARRIVE guidelines recommended for reporting animal research

Laboratory animals	Male and female C57BL6/J mice up to 14 months of age were used in this study. Mice were housed at 72 degree Fahrenheit under a 12-hour light/12-hour dark cycle and maintained on a standard chow diet. Mice had ad libitum access to food and water. To obtain myofiber-restricted deletion of Mettl3, Mettl3 LoxP-targeted (flox; fl) mice (Mettl3fl/fl) crossed to mice expressing tamoxifen-inducible Cre recombinase gene under the control of the skeletal myofiber-specific human skeletal alpha actin (HSA) promoter to generate HSA-MerCreMer (M3-mKO) mice. Mice that were wild-type for Mettl3, but expressing Cre recombinase or the flox allele without Cre were used as controls. To generate mice with myofiber specific HA-tagged ribosomes, M3-mKO or HSA expressing controls were crossed with homozygous RiboTag34 (RPL22HA, #011029, The Jackson Laboratory) mice.
Wild animals	No wild animals were used in this study.
Field-collected samples	No field-collected samples were used in this study.
Ethics oversight	Animal care and use procedures were performed in accordance with the standards set forth by the Institutional Animal Care and Use Committee at The Ohio State and the Guide and Care and Use of Laboratory Animals published by the US National Institute of Health; all protocols were approved by The Ohio State University Institutional Animal Care and Use Committee.

Note that full information on the approval of the study protocol must also be provided in the manuscript.

This checklist template is licensed under a Creative Commons Attribution 4.0 International License, which permits use, sharing, adaptation, distribution and reproduction in any medium or format, as long as you give appropriate credit to the original author(s) and the source, provide a link to the Creative Commons license, and indicate if changes were made. The images or other third party material in this article are included in the article's Creative Commons license, unless indicated otherwise in a credit line to the material. If material is not included in the article's Creative Commons license and your intended use is not permitted by statutory regulation or exceeds the permitted use, you will need to obtain permission directly from the copyright holder. To view a copy of this license, visit http://creativecommons.org/licenses/by/4.0/
